# Effect of Rodent Control Program on Incidence of Zoonotic Cutaneous Leishmaniasis, Iran

**DOI:** 10.3201/eid3007.231404

**Published:** 2024-07

**Authors:** Amir Abdoli, Samaneh Mazaherifar, Kavous Solhjoo, Mohsen Farhang Zargar, Hayedeh Parvin Jahromi, Ali Taghipour, Mohammad Darayesh, Milad Badri, Majid Pirestani, Shahab Falahi, Azra Kenarkoohi

**Affiliations:** Jahrom University of Medical Sciences, Jahrom, Iran (A. Abdoli, S. Mazaherifar, K. Solhjoo, M. Farhang Zargar, H.P. Jahromi, A. Taghipour, M. Darayesh);; Qazvin University of Medical Sciences, Qazvin, Iran (M. Badri);; Tarbiat Modares University, Tehran, Iran (M. Pirestani);; Ilam University of Medical Sciences, Ilam, Iran (S. Falahi, A. Kenarkoohi)

**Keywords:** Cutaneous leishmaniasis, *Leishmania major*, zoonoses, rodent control program, parasites, Iran

## Abstract

We report the effect of a rodent control program on the incidence of zoonotic cutaneous leishmaniasis in an endemic region of Iran. A 1-year interruption in rodent control led to 2 years of increased incidence of zoonotic cutaneous leishmaniasis. Restarting rodent control led to a decline of zoonotic cutaneous leishmaniasis.

Leishmaniasis is a neglected tropical disease that is prevalent worldwide (*1*). Cutaneous leishmaniasis (CL) is endemic in different regions of Iran and has an approximate incidence rate of 1.18 cases/100,000 population and a difference of 5.7 disability-adjusted life-years (*2*). Zoonotic cutaneous leishmaniasis (ZCL), caused by the parasite *Leishmania major*, is the primary cause of CL in Iran, where anthroponotic cutaneous leishmaniasis, caused by *Leishmania tropica*, is less prevalent (*2*,*3*). Because rodents are the main reservoirs of ZCL, the rodent control program (RCP) is an important intervention for the control of ZCL in endemic areas (*1*). ZCL is endemic in Jahrom county in Fars Province, Iran (Appendix Figure 1). We report the effects of an RCP on incidence of ZCL in this region. This study was approved by the Research and Ethics Committee of Jahrom University of Medical Sciences, Jahrom, Iran (ethics code IR.JUMS.REC.1402.067).

## The Study

The RCP is conducted in rural areas of Jahrom county 5 times a year, beginning with the destruction of rodent nests in early April (*4*). Once a month in April, May, June, and September, rodent nest baiting is performed by using a mixture of wheat with 2.5% zinc phosphide (*4*) (Appendix Figure 3). The rodent nest baiting is conducted in a 500-m circle around houses within the intervention area (*4*). 

The RCP was completely ceased during the first year of the COVID-19 pandemic in Jahrom county because of a lack of equipment and personnel. The program was resumed routinely in early April 2021 (*5*). The outcome of the 1-year interruption in the RCP was a mild increase in the incidence of ZCL in 2020 and a high increase in ZCL in 2021 (Appendix Figure 2). Incidence rates for the period before the pandemic were stable: 103.7 cases/100,000 population in 2016, 95.1 cases/100,000 population in 2017, 99.7 cases/100,000 population in 2018, and 99.6 cases/100,000 population in 2019 (Figure). After the RCP stopped because of the COVID-19 pandemic, ZCL incidence rates increased to 129.4 cases/100,000 population in 2020 and 321.5 cases/100,000 population in 2021, (Table; Figure). Of interest, the outcome of restarting the RCP in 2021 was not apparent until 2022 and 2023, when the incidence rate of ZCL decreased to 72.1 cases/100,000 population in 2022 and 19.2 cases/100,000 population in 2023 (Table; Figure; Appendix Figure 2).

**Figure:**
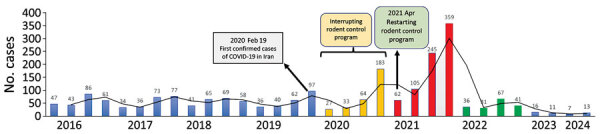
Trends of cutaneous leishmaniasis from 2016 to the beginning of 2024 in Jahrom county, Iran, highlighting the period during the COVID-19 pandemic in which the rodent control program was interrupted in 2020 and restarted in 2021.

**Table:** Annual incidence rate of zoonotic cutaneous leishmaniasis cases/100,000 people in Jahrom county, Iran, between 2016 and 2023.

Year	Total population	No. patients	Incidence rate (per 100,000)
2016	228,443	237	103.7
2017	231,256	220	95.1
2018	233,592	233	99.7
2019	235,825	235	99.6
2020	237,194	307	129.4
2021	239,829	771	321.5
2022	242,852	175	72.1
2023	244,458	47	19.2

Jahrom county has an agricultural environment that provides suitable conditions for both the rodent reservoirs and sand fly vectors of *L. major*. Although different studies have demonstrated the effectiveness of the RCP for control of ZCL (*4*,*6*,*7*), little is known about the impact of a short-term interruption of the RCP on the incidence of ZCL in an endemic region. The unplanned interruption of the RCP in Jahrom county because of the COVID-19 pandemic gave us the opportunity to evaluate the impact of the RCP on the incidence of ZCL in an endemic area. Although the RCP was paused for only 1 year and resumed routinely in early April 2021, a slight increase in the incidence of ZCL was observed (129.4 cases/100,000 population in 2020) and a marked increase in ZCL the following year (321.5 cases/100,000 population in 2021). However, because of the COVID-19 lockdown, the diagnosis and treatment of CL patients were interrupted during the first year of the pandemic (2020), so those factors could be involved in the increase in the incidence of ZCL in 2020 and 2021. Of interest, a sharp decline in the incidence rates was observed in 2022 and 2023 (72.1 cases/100,000 population in 2022 and 19.2 cases/100,000 population in 2023). However, comparing the incidences of ZCL in 2022 and 2023 with the incidences before 2020 has shown that factors other than the RCP could be contributing to the decline of ZCL because the incidence rates of 2022 and 2023 were much lower than the incidence rates before 2020 (Table; Figure; Appendix Figure 2). Other factors, such as climate conditions and rainfall, which are involved in the propagations of rodents and sand flies, could also contribute to the decline of ZCL observed in 2022 and 2023.

The emergence of COVID-19 has a considerable influence on the burden of noncommunicable and communicable diseases throughout the world and in Iran (*8*,*9*). In Brazil, the COVID-19 pandemic seems to have contributed to an increase in the incidence of tegumentary leishmaniasis in 2020 (*10*). A decreasing incidence of CL was reported in an endemic region in western Iran during the COVID-19 pandemic (2020–2021), possibly because of the disruption of CL diagnosis and treatment follow-up (*11*).

## Conclusions

Our study showed a 1-year interruption to the RCP contributed to 2 years of increased incidence of ZCL in an endemic region of Iran. Restarting the RCP led to the decline of ZCL after 2 years of program activity. However, additional factors, such as environmental conditions (e.g., climate conditions and rainfall) that have an influence on vector and reservoir propagation, and diagnosis and treatment follow-up, could influence the incidence of ZCL (*12*, *13*). In the absence of a vaccine for ZCL, an RCP is an effective strategy for ZCL control in endemic regions.

Appendix Additional information about effect of rodent control program on incidence of zoonotic cutaneous leishmaniasis, Iran
