# Supplementary material for: Effect of Rodent Control Program on Incidence of Zoonotic Cutaneous Leishmaniasis, Iran
Source: Emerg Infect Dis. 2024 Jul;30(7):1447–9. doi: 10.3201/eid3007.231404 (PMC11210642; doi:10.3201/eid3007.231404)
Supplement: Appendix — Additional information about effect of rodent control program on incidence of zoonotic cutaneous leishmaniasis, Iran [file 23-1404-Techapp-s1.pdf]

Article DOI: https://doi.org/10.3201/eid3007.231404

EID cannot ensure accessibility for supplementary materials supplied by authors. Readers who have difficulty accessing supplementary content should contact the authors for assistance.

Effect of Rodent Control Program on Incidence of Zoonotic Cutaneous Leishmaniasis, Iran

Appendix

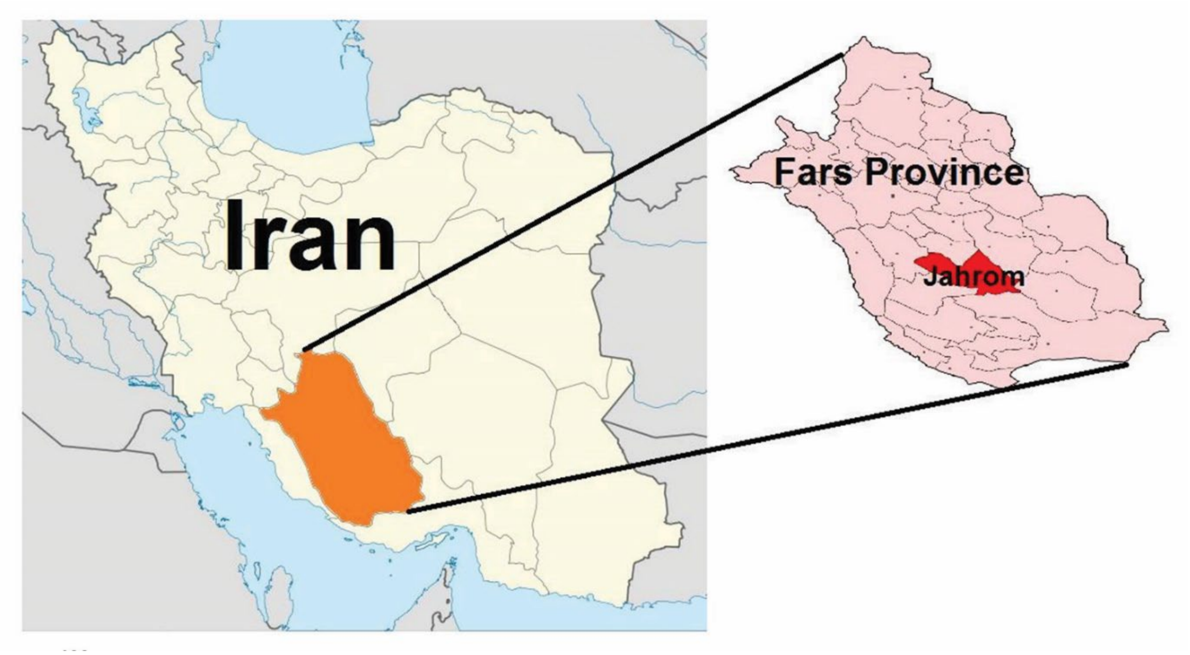

Appendix Figure 1. The map of Iran and the outbreak region (Jahrom, Fars Province).

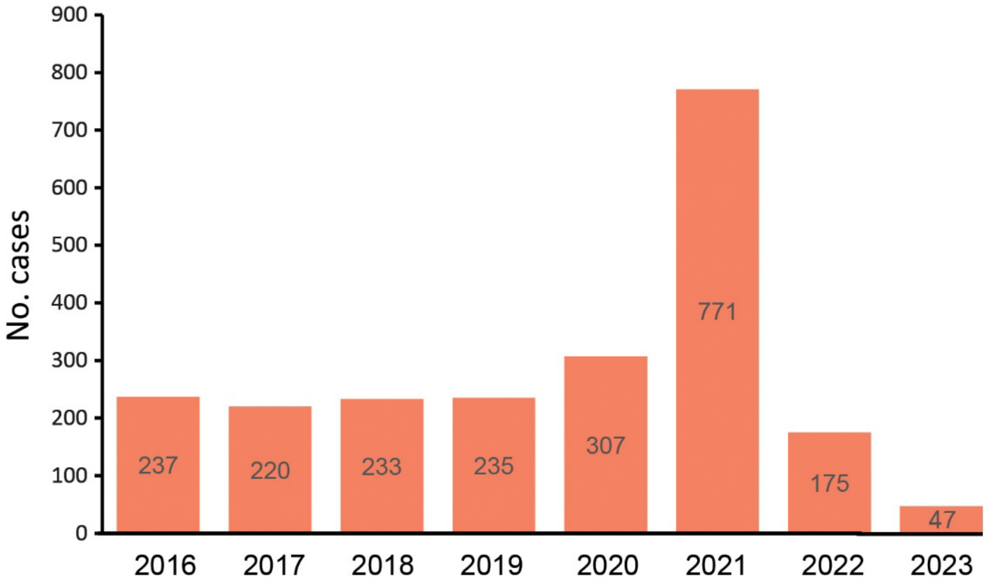

Appendix Figure 2. Yearly trends (number of cases) of cutaneous leishmaniasis occurrence in Jahrom, Iran from 2016 to 2022.

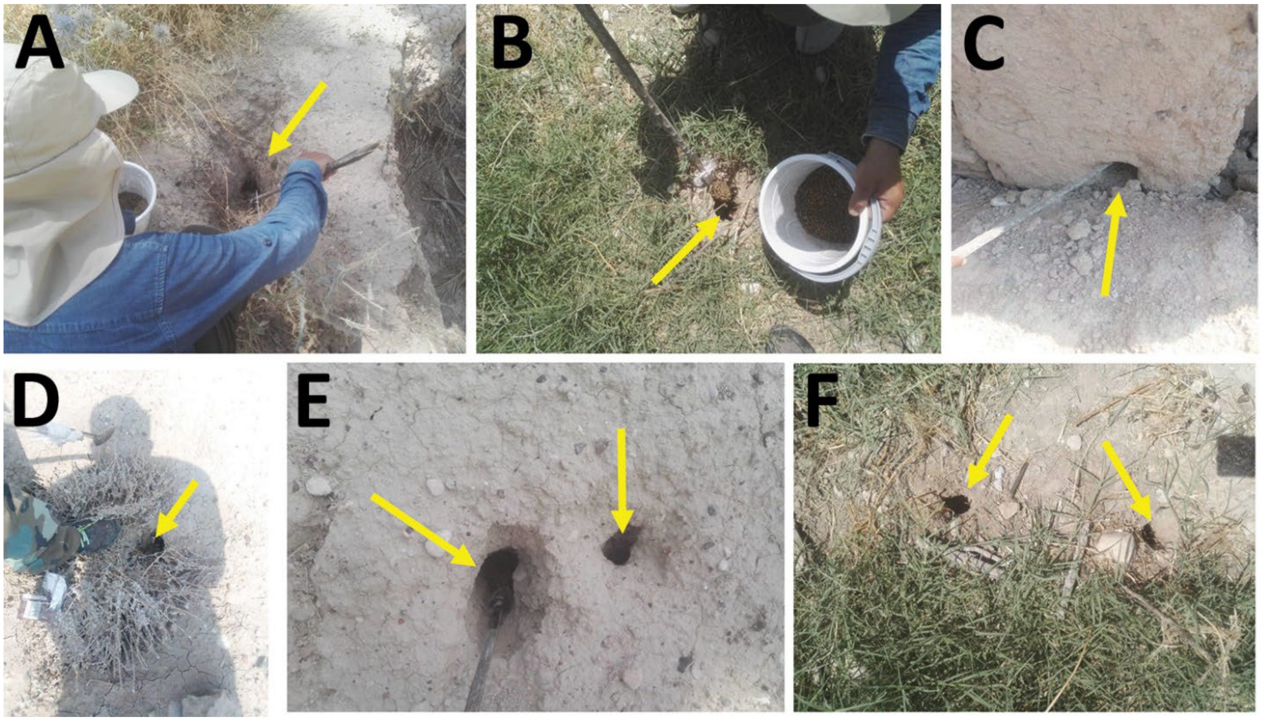

Appendix Figure 3. Rodent nest baiting using a mixture of wheat with 2.5% zinc phosphide. Yellow arrows indicate the entrance to rodent nests.
